# Surgical treatment of obesity

**DOI:** 10.12688/f1000research.13515.1

**Published:** 2018-05-21

**Authors:** Vance L. Albaugh, Naji N. Abumrad

**Affiliations:** 1 Department of Surgery, MCN CC-2308, Section of Surgical Sciences, Vanderbilt University Medical Center, Nashville, Tennessee, USA

**Keywords:** metabolic surgery, bariatric surgery, diabetes, weight loss

## Abstract

Obesity prevalence continues to increase worldwide, as do the numerous chronic diseases associated with obesity, including diabetes, non-alcoholic fatty liver disease, dyslipidemia, and hypertension. The prevalence of bariatric surgery also continues to increase and remains the most effective and sustainable treatment for obesity. Over the last several years, numerous prospective and longitudinal studies have demonstrated the benefits of bariatric surgery on weight loss, mortality, and other chronic diseases. Even though the mechanisms underlying many of these beneficial effects remain poorly understood, surgical management of obesity continues to increase given its unmatched efficacy. In this commentary, we discuss recent clinical advancements as well as several areas needed for future research, including indications for bariatric and metabolic surgery, determination of responders and non-responders, metabolic surgery in non-obese individuals, and the evolving role of bariatric surgery in adolescents.

## Introduction

Obesity prevalence continues to rise and has become the most significant disease affecting health care worldwide ^1^. Not only does obesity have close associations with diabetes and cardiovascular disease ^1– 3^ but it is also a risk factor for cancer ^4^ and non-alcoholic fatty liver disease (NAFLD) that can progress to cirrhosis and liver failure ^5, 6^. The burden of obesity on quality of life as well as the economy ^7– 9^ has spurred the development of numerous weight loss therapies that range from behavioral to pharmacologic to surgical.

The management of obesity has become considerably more complex as our understanding of weight regulation has also increased. Genetic studies suggest that body weight is at least partially heritable, with heritability estimates ranging from 40% to 70% and differing significantly between the sexes ^10^. Clearly, there are some well-defined monogenic forms of obesity ^11^, but for the great majority of overweight or obese individuals, environment drives the accumulation and maintenance of body weight over time. Diet, exercise, and other lifestyle interventions have failed to lead to robust and sustainable weight loss ^12– 14^. Moreover, isolated pharmacologic therapies targeting body weight regulation have insufficient effect sizes. To date, bariatric surgery is the only effective therapy that leads to marked and sustained body weight loss.

Why is bariatric surgery so effective against obesity? How does bariatric surgery lead to these sustained effects? These questions remain despite an increasingly complex understanding of bariatric surgery and its postoperative physiology ^15, 16^. A likely explanation is that body weight regulation is such a highly regulated process that targeting an isolated hormonal or neural pathway pharmacologically is easily overridden by a multitude of other factors contributing to weight maintenance. This physiology means that lifestyle interventions (for example, exercise and dietary modifications) and other pharmacologic approaches undoubtedly fail with time. If one is able to lose weight in the short term, then he or she is continually fighting the natural homeostatic processes attempting to counteract that degree of weight loss. Unlike non-surgical interventions, however, bariatric surgery concurrently affects multiple anatomic and physiologic processes that are arguably impossible to collectively target pharmacologically. Numerous basic and clinical studies have identified a variety of observations, including augmented secretion of satiety factors from the gastrointestinal tract ^17– 19^, altered neural circuitry in the gut and brain ^15, 20– 23^, remodeling of the gut microbiome ^24– 27^, altered gastric emptying ^28, 29^, rapid intestinal nutrient delivery ^30^, and (probably) more ^31^. Overall, bariatric surgery targets a variety of pathways involved in body weight regulation which enable it to exert powerful and sustained effects.

As bariatric surgery continues to grow and surgical treatment of obesity and other chronic illnesses (for example, diabetes, dyslipidemia, and hypertension) continues to rise, understanding the short- and long-term effects and outcomes of these operations will become even more important. In the following, we briefly review the current surgical treatment of obesity and recent evidence demonstrating its efficacy, not only for obesity but also for other chronic illnesses, and the future directions and questions the field will face in the coming decade.

## Bariatric surgery

The prevalence of bariatric surgery continues to increase across the globe ^32, 33^, although the rate of increase is slowing in North America. The types of bariatric operations and other procedures being performed are also continuing to rise. Aside from evolving experimental operations (for example, one-anastomosis/single-anastomosis gastric bypass and gastric plication), there are several novel types of endoscopic interventions in preclinical or clinical testing (for example, gastric balloons and duodenal mucosal resurfacing). Obesity treatment is an active area of research, and, given the breadth of emerging devices, we are focusing on only the most common operations herein. Regardless, experimental operations and devices do not yet contribute to a significant number of procedures worldwide, but these evolving tools may play an increasing role in obesity management in the future.

**Figure 1.**
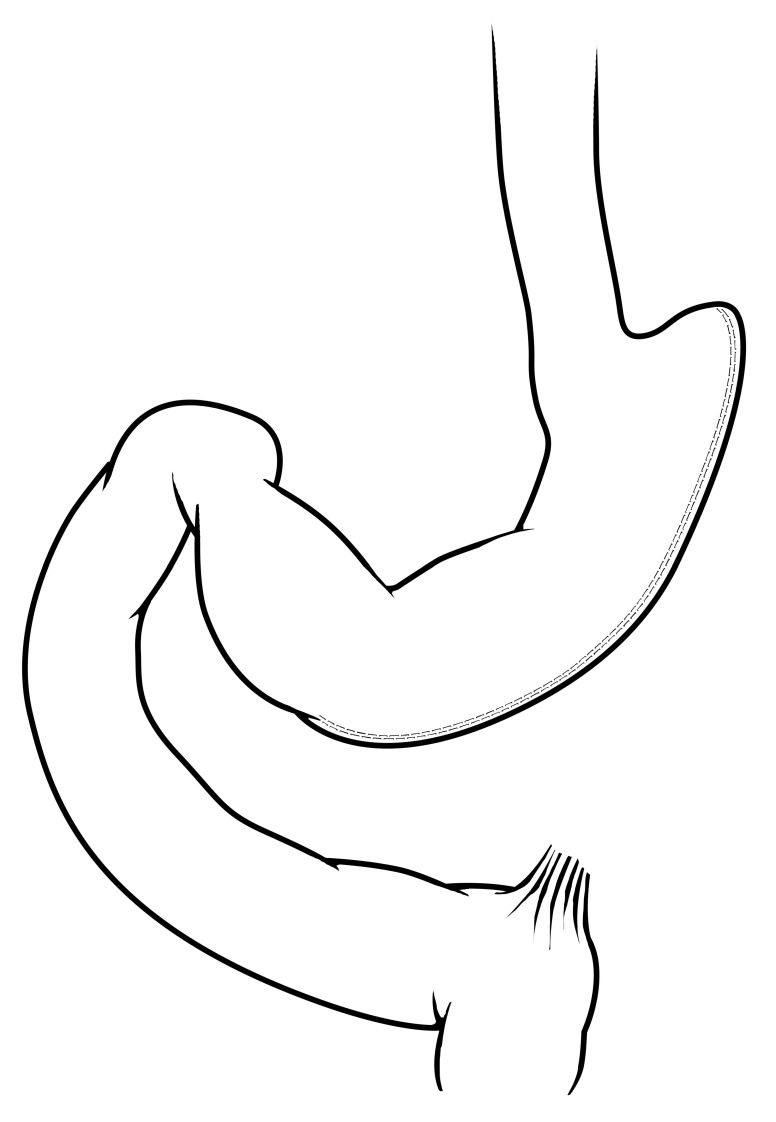
Vertical sleeve gastrectomy. A majority of the greater curvature of the stomach is excised in this operation, creating a tube-like stomach with a marked reduction in gastric capacity. Reprinted with permission ^35^.

In terms of surgery, three operations make up the overwhelming majority of bariatric surgical volume worldwide. These include the vertical sleeve gastrectomy (VSG), Roux-en-Y gastric bypass (RYGB), and adjustable gastric banding (AGB). The VSG ( Figure 1) is the most popular bariatric operation worldwide and is estimated to account for nearly 50% of all operations ^32^. VSG is performed by using a cutting/sealing tissue stapler to create a long stomach tube that resembles a “sleeve”, irreversibly removing the greater curvature of the stomach. The greater curvature of the stomach is a known site of secretion for ghrelin and other gastrointestinal hormones ^34^. There is no other gastrointestinal rearrangement with the VSG operation. In general, VSG is well tolerated and, like all bariatric operations, has a very low rate of perioperative complications (<1%) in experienced hands. Studies typically report a weight loss of between 50% and 60% of excess body weight ^36, 37^, and excess body weight is calculated from ideal body weight ^38^.

**Figure 2.**
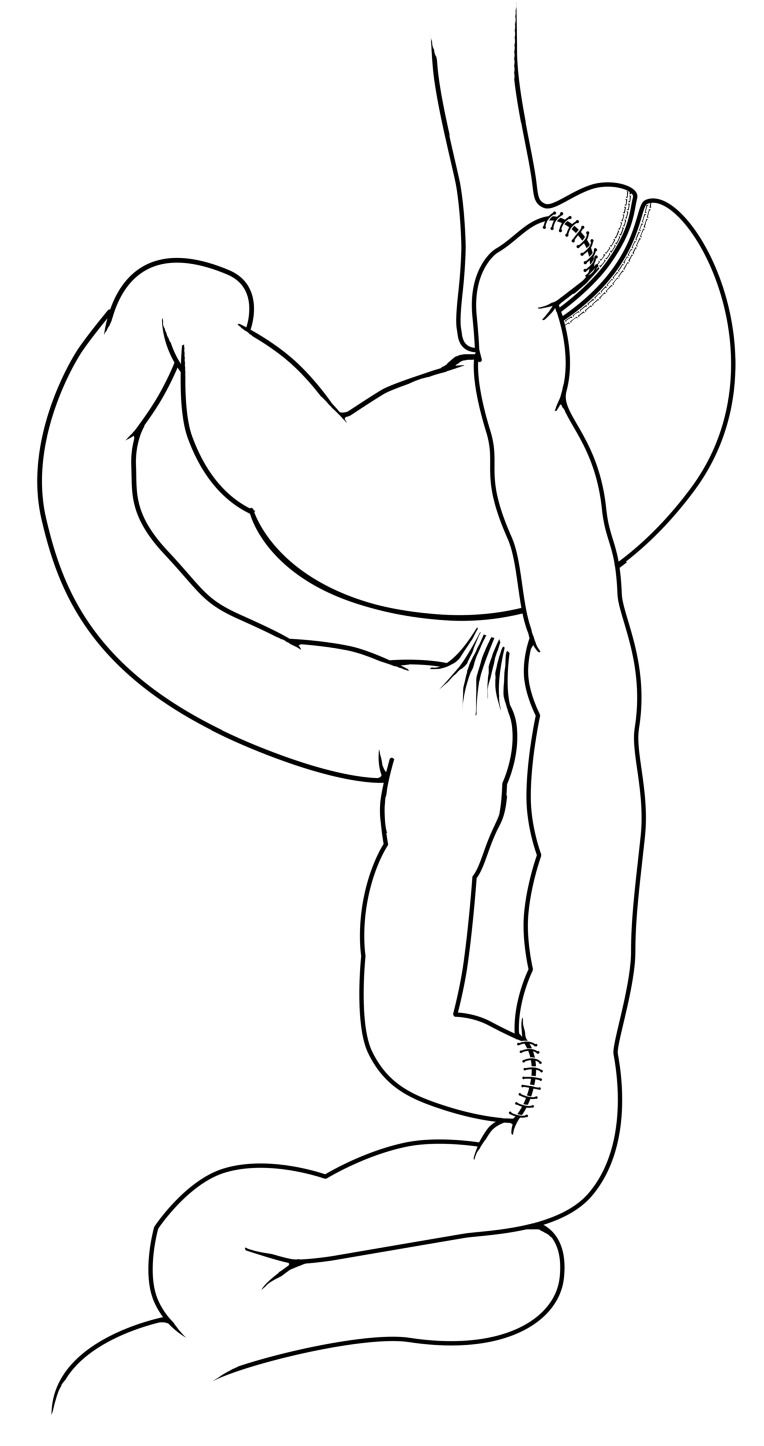
Roux-en-Y gastric bypass. The stomach is divided, creating a small gastric pouch (about 30 mL) that is connected via a gastrojejunostomy to a distal segment of jejunum, which forms the Roux limb of the procedure. The remainder of the stomach is referred to as the “gastric remnant” and drains into the bypassed portion of bowel, referred to as the “biliopancreatic limb”. Bowel continuity is restored for the biliopancreatic limb by a jejunojejunostomy, creating the “Y” configuration of the operation. Thus, ingested nutrients proceed rapidly through the stomach pouch and move immediately into the jejunal Roux limb in the absence of bile and pancreatic secretions. Bile and pancreatic secretions drain via the biliopancreatic limb and then mix with the chyme/nutrients at the point of the jejunojejunostomy. Reprinted with permission ^35^.

RYGB ( Figure 2) is the second most prevalent bariatric operation worldwide and is estimated to contribute to about 40% of bariatric operations ^32^. RYGB was formerly the most prevalent operation until recently surpassed by VSG. Surgically, RYGB is technically more challenging, as it involves creating a small stomach pouch (typically about 30 mL) that is connected to an end of the more distal small intestine (that is, jejunum), which creates a “Roux” limb (about 100–150 cm). This Roux limb is sometimes referred to as the alimentary limb, the limb by which foodstuffs travel after transit through the stomach pouch. In order to re-establish the flow of biliary and pancreatic digestive secretions from the liver and pancreas, respectively, the excluded limb of bowel is connected downstream to meet the Roux limb. This limb carrying bile and pancreatic enzymes is referred to as the biliopancreatic limb (about 50–75 cm). The convergence of the Roux and biliopancreatic limbs is connected at the jejunojejunostomy and forms a Y-configuration. The two limbs join at this site, and the remaining distal small bowel is known as the common channel. The common channel is the only site for mixing of digestive enzymes/secretions from the biliopancreatic limb with foodstuffs of the Roux limb. Unlike VSG that has one long staple line forming the sleeve-like stomach, the RYGB has two anastomoses or “connections” created during the operation as well as the stomach remnant that remains in place to drain gastric secretions into the biliopancreatic limb. Even despite these two connections, the risk of intestinal leak or bleeding occurs infrequently in the perioperative setting (<1%) ^39, 40^. In terms of weight loss, studies have demonstrated that weight loss of RYGB is similar to VSG, reaching about 50–60% of excess body weight loss ^36, 37^.

**Figure 3.**
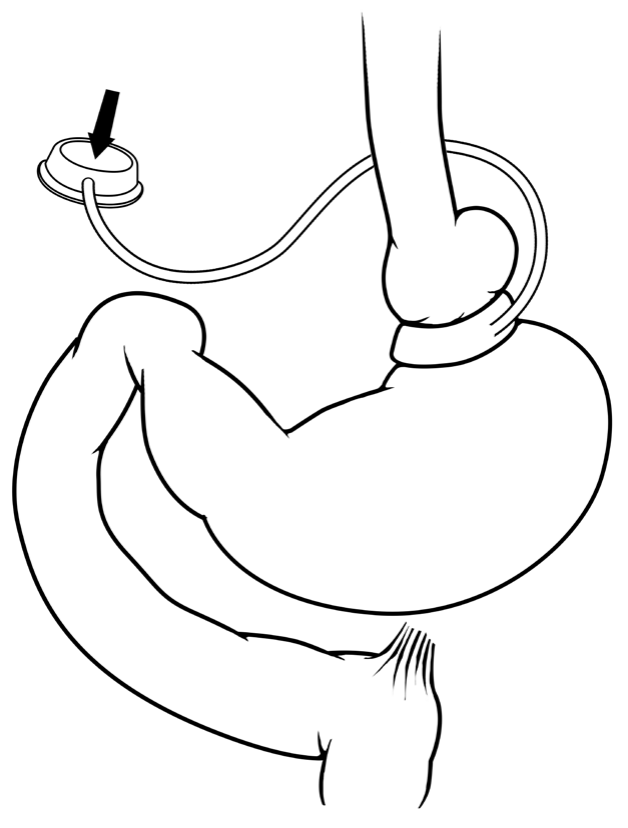
Adjustable gastric banding. In this operation, an external ring is placed around the proximal portion of the stomach and has a balloon that lines the inside portion of the ring. The inflatable balloon is connected to a port in the subcutaneous tissue of the upper abdomen that allows the balloon volume, and therefore the amount of external gastric restriction, to be adjusted. Reprinted with permission ^35^.

An operation that has fallen out of favor for obesity management is the AGB ( Figure 3), although it is estimated to continue to contribute to approximately 7% of bariatric operations ^32^. The banding operation involves placing an externally compressive device on the upper portion of the stomach, which can be inflated or deflated with a subcutaneous port, permitting adjustment of the degree of gastric compression to limit stomach distention and food intake. Even though this is the third most common procedure and has the benefit of being completely reversible, its efficacy pales in comparison with other bariatric operations. Originally, the promise of the AGB was fewer complications but preserved weight loss efficacy. Even though perioperative complications associated with AGB are also rare (<1%), the lack of efficacy and the advent of newer and more effective options in the surgical armamentarium have led to increasingly fewer individuals choosing AGB. The weight loss response with AGB is highly variable, and prospective studies show, on average, a body weight loss of about 20% ^41^. Given the trends in AGB over the last decade, its use will likely continue to fall, especially with the increasing use of other non-surgical procedures.

**Figure 4.**
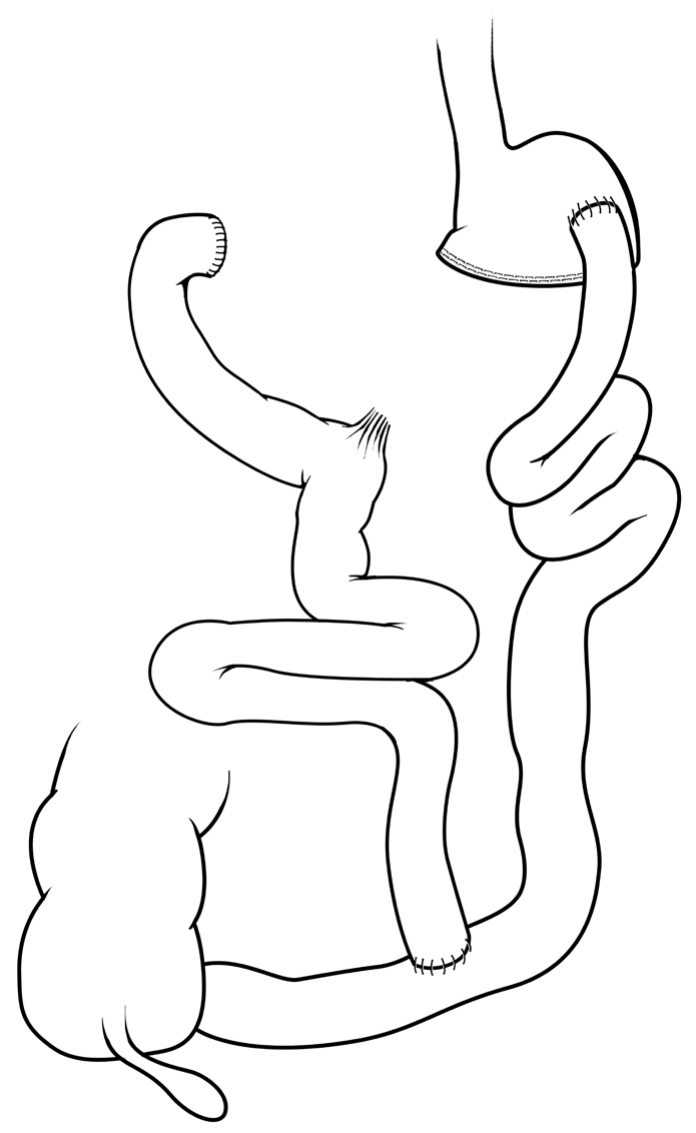
Biliopancreatic diversion. This operation essentially diverts bile and pancreatic secretions to the distal bowel for mixing with nutrients/chyme, typically much further distal than a Roux-en-Y gastric bypass. Traditional biliopancreatic diversion consists of a modest reduction in stomach volume, typically about 300–400 mL, as well as the diversion of bile and pancreatic secretions. Reprinted with permission ^35^.

**Figure 5.**
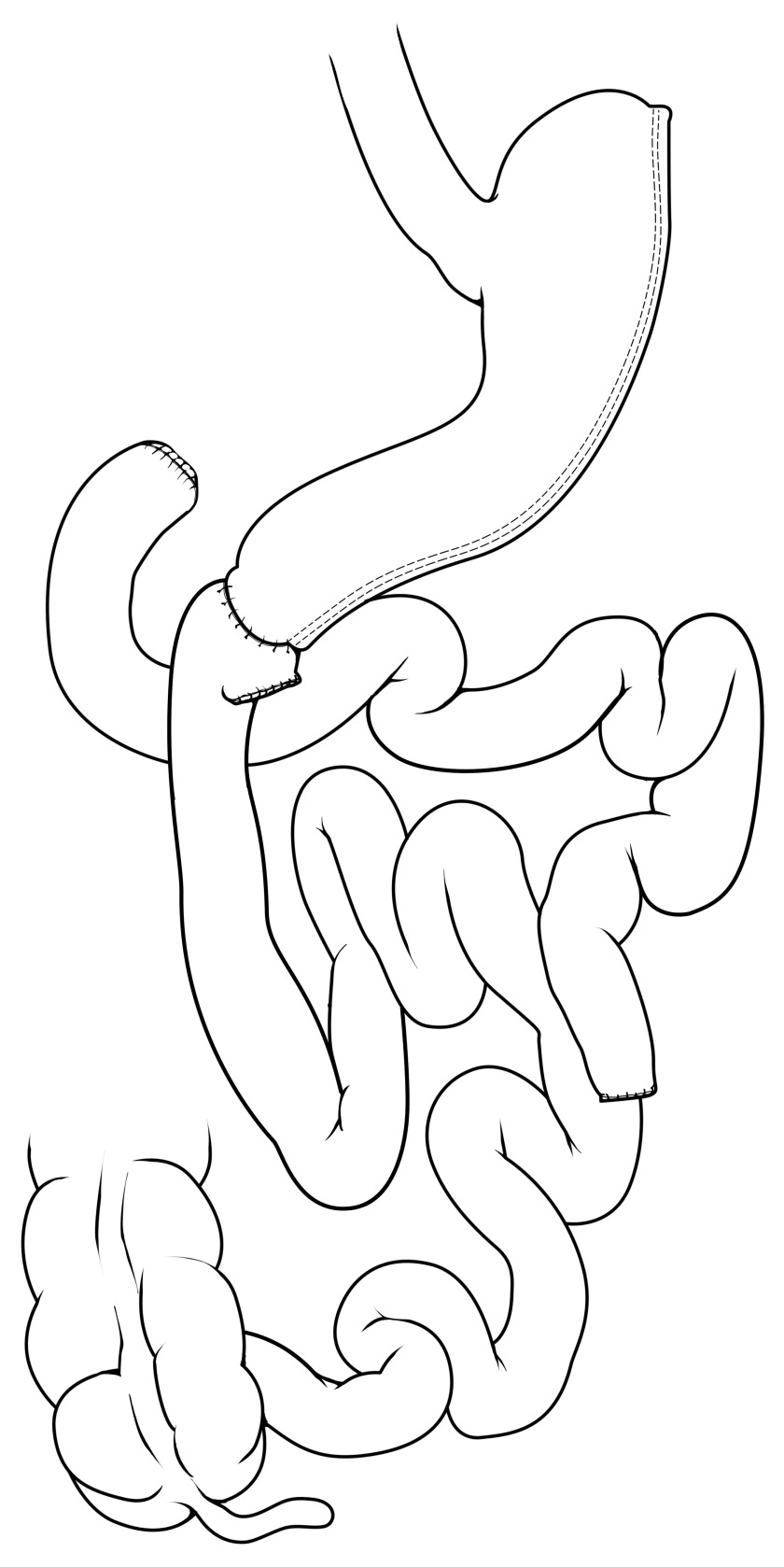
Biliopancreatic diversion with duodenal switch. As in traditional biliopancreatic diversion, bile and pancreatic secretions are diverted to the distal bowel for mixing with nutrients/chyme. The duodenal switch component, however, is accompanied by a vertical sleeve gastrectomy for reduction of gastric volume.

Biliopancreatic diversion (BPD) and BPD with duodenal switch (BPD/DS) are two less commonly performed operations worldwide (~1% overall). However, similar to RYGB, these operations involve significant rearrangement of the small intestines with a gastric resection that leaves either a smaller stomach pouch (about 300–400 mL) with the BPD ( Figure 4) or a sleeve-like stomach with the BPD/DS ( Figure 5). These are similar operations, but in each case the biliopancreatic secretions are diverted far distal (typically about 100–150 cm proximal to the colon). Compared with RYGB, BPD and BPD/DS have improved weight loss efficacy ^42^, and estimates are around 60–70% of excess body weight loss. Even though this has not been well studied, in limited larger studies this efficacy is at the expense of increased perioperative morbidity ^43^ as well as nutritional complications (for example, vitamin and mineral deficiencies) that are associated with a small but significant increase in daily bowel movements consistent with fast intestinal transit and malabsorption ^42^.

## Bariatric surgery and clinical outcomes

### Weight loss and mortality benefits

Even though bariatric surgery dates to the 1960s ^44^, the last decade has seen a number of rigorous, high-quality studies focused on the effects of surgical weight loss and associated chronic medical conditions. The first and largest prospective case controlled study examining surgical weight loss was, and remains, the Swedish Obesity Subjects (SOS) study ^45, 46^. Despite being overrepresented with vertical banded gastroplasty, an operation that has since fallen out of favor, the SOS study was the first prospective study demonstrating superior weight loss with bariatric surgery (all operations grouped together) compared with non-surgical alternatives ^47^. More recently, other long-term studies have confirmed that bariatric surgery leads to significantly greater weight loss compared with non-surgical interventions. A retrospective cohort of Veterans Affairs patients (n = 1,787) showed that RYGB led to significantly greater and sustained weight loss compared with VSG or AGB. RYGB led to about 10% more weight loss (relative to baseline body weight) compared with VSG and about 17% more compared with AGB ^48^. Similar results were seen in observational studies by Adams and colleagues, showing about 28% weight loss (relative to baseline body weight) compared with 0.2% and 0% in non-surgical patients ^49^. Longer observational studies (>10 years) have corroborated these estimates and demonstrated superior weight loss with surgery at 10 and 12 years ^50, 51^ that is unmatched by non-surgical therapy.

Even though retrospective epidemiological studies suggested that weight loss might be associated with increased mortality ^52– 54^, the prospective and randomized Look AHEAD (Action for Health in Diabetes) trial demonstrated that weight loss secondary to intensive lifestyle intervention was not associated with increased mortality. However, the weight loss in the intensive lifestyle group compared with controls at the end of the study (6% versus 3.5%) was also not associated with decreased mortality ^55^. Unlike weight loss secondary to intensive lifestyle intervention, weight loss secondary to bariatric surgery has been shown to be associated with decreased mortality. Ten-year estimates from the SOS study ^56^ show significant decreases in mortality risk (adjusted hazard ratio of 0.71). These prospective, observational findings from the SOS study have also been demonstrated in a retrospective cohort of RYGB with cause-specific mortality rates decreased by 56% for coronary artery disease, 92% for diabetes, and 60% for cancer ^57^. Even though the majority of bariatric surgery is done in women, similar mortality and cardiovascular benefits are also observed in men ^58^ but with lower cardiovascular events and cardiovascular-related death ^59^. RYGB and VSG have the best-studied effects overall for weight loss, although AGB has also shown decreased 5- and 10-year all-cause mortality, a benefit that is observed in both sexes ^58^.

### Perioperative and long-term safety

Although surgical treatment of obesity is the most effective therapy, it is also the most invasive with perioperative risk related to the operation itself, including general anesthesia and postoperative recovery. As bariatric surgery has continued to become more prevalent, there have been concerns raised about the perioperative safety of these procedures. Improved anesthetic and surgical management of obese patients undergoing bariatric surgery, however, has been shown to have no greater risk than other elective operations routinely performed in adults and pediatric patients ^60– 62^, including laparoscopic cholecystectomy and laparoscopic hysterectomy ^63^. Estimates for perioperative 90-day mortality for bariatric surgery are approximately 0.3%, and quality improvement programs are continuing to work to make these low numbers even lower.

Aside from perioperative safety, which has been well studied, a number of smaller prospective, randomized trials demonstrated that bariatric surgery appears to be safe overall at least up to five years postoperatively ^40, 64^, although these trials are not without limitations. Studies to date ^40, 64^ have shown that surgery is associated with adverse events that one would expect to be more prevalent following surgery (for example, bowel obstruction). Given the difficulties of longer-term follow-up (>5 years), complications associated with surgical management (for example, recurrent bowel obstruction, bleeding, perforation, and marginal ulceration) are not well studied and represent an important area for future study. Recent retrospective studies in smaller cohorts suggest that these types of adverse events related to surgery are not insignificant and the long-term benefits and risks associated with surgery should be considered given the degree of obesity and other comorbidities when a patient is considered for surgery ^65^.

### Resolution of metabolic diseases

In addition to the clear benefits associated with weight loss, one of the most intriguing effects of bariatric surgery is its tendency to resolve other chronic metabolic diseases (for example, diabetes and dyslipidemia) *prior to* weight loss. A substantial portion of these changes preceding significant weight loss are driven by caloric restriction perioperatively, as the effects of dietary restriction are well known ^66, 67^ and have been examined in patients following bariatric surgery ^68– 71^. Regardless, there are other detectable metabolic effects that occur independently of caloric restriction (for example, 72– 74). The role of these metabolic effects in the short- and long-term metabolic outcomes of these operations is not well understood.

Even though these effects occur prior to significant weight loss (reviewed in 16, 35, 75), postoperative weight loss undoubtedly further improves these chronic disease states that are exacerbated by obesity. To better understand these effects related to bariatric surgery, several randomized and prospective studies to date have targeted these effects on metabolic diseases (for example, diabetes and dyslipidemia) and their response over time. Surgical Treatment And Medications Potentially Eradicate Diabetes Efficiently (STAMPEDE) is a prospective, randomized trial demonstrating that bariatric surgery (RYGB and VSG) is more effective than intensive lifestyle therapy alone for diabetes treatment and has sustained benefits, including weight loss up to 5 years ^76^. To date, both RYGB and VSG have similarly improved diabetes efficacy, even though RYGB tends to have slightly increased weight loss. The Diabetes Surgery Study (DSS) is another 5-year, randomized, observational study examining RYGB added to intensive lifestyle therapy and medical management of type 2 diabetes. The DSS has the additional benefits of examining a triple endpoint—that is, systolic blood pressure of less than 130 mmHg, hemoglobin A1C of less than 7%, and low-density lipoprotein (LDL) cholesterol of less than 100 mg/dL—based on optimal diabetes management guidelines as well as having multiple, including international, study sites ^64^. Similar to STAMPEDE, the DSS showed a significant benefit of added RYGB to intensive lifestyle and medical management to the triple endpoint at 5 years, although the effect appeared to wane over time. Both DSS and STAMPEDE are similar to a third trial—conducted by Mingrone and colleagues—examining RYGB and BPD for weight loss and diabetes management, demonstrating that degree of weight loss is not necessarily predictive of which patients will have diabetes resolution ^77^. This third trial has the smallest sample size—three groups of 20 subjects per group (that is, intensive medical treatment, RYGB, BPD)—a clear limitation compared with STAMPEDE that allocated 50 subjects to three groups (that is, intensive lifestyle/medical, RYGB, and VSG) and the DSS with 120 subjects overall in two equal groups at randomization (that is, intensive lifestyle/medical and RYGB).

Overall, it is important to note that the clinical trials mentioned above ^40, 64, 77^ as well as other studies demonstrate that the benefits of RYGB on diabetes resolution are not limited to class III obese subjections (that is, body mass index [BMI] of more than 40 kg/m ^2^) ^78^. Even in mild to moderate obesity (BMI of 30–39.9 kg/m ^2^), RYGB not only leads to superior diabetes resolution or improvement compared with medical therapy (28% versus 0%) but also helps patients meet other biochemical goals of diabetes management (for example, hemoglobin A1C, LDL cholesterol, and systolic blood pressure) ^64^. Even though most studies focus on the resolution of insulin resistance/diabetes, effects on other cardiovascular markers (that is, LDL cholesterol and blood pressure) are important and, though less commonly observed, represent an area for further study. Aside from using bariatric surgery as a treatment for diabetes, the SOS study ^79^ examined the role of surgery for the prevention of diabetes with impressive results (adjusted hazard ratio of 0.17).

Even though all patients may not have complete resolution of diabetes, data suggest that diabetes and insulin resistance are significantly ameliorated. Aside from the trials discussed above, a single-center trial (n = 69 overall) comparing RYGB, AGB, and lifestyle intervention for diabetes showed 3-year follow-up data with either partial or complete resolution of diabetes in 40% of RYGB and 29% of AGB compared with zero in a lifestyle intervention group. Consistent with these rates, even in those without complete resolution, the use of diabetes medications decreased in the surgical group (−65% with RYGB and −33% with AGB) compared with none in the lifestyle weight loss group ^80^. Overall, evidence indicates that these operations have significant effects on weight loss as well as benefits to metabolic disease.

### Cancer

Unlike the seemingly more direct relationship between obesity and atherosclerosis or diabetes, the relationship between cancer and obesity remains intriguing. Obesity is a bona fide risk factor for cancer ^3, 4^, with protective effects conferred by bariatric surgery, an effect that is presumably due to weight loss over time associated with surgery ^57, 81, 82^. It should be noted that the mortality benefit of bariatric surgery from the SOS study mentioned above ^56^ is driven primarily by decreased cancer-related death, more so than major cardiovascular outcomes. The links between bariatric surgery and cancer are strong, and weight management and obesity treatment using bariatric surgery to decrease cancer risk as well as cancer recurrence are gaining popularity among oncologists ^83^. The interaction of bariatric surgery and cancer is an intense area of investigation, from not only an epidemiological perspective but also a basic scientific perspective. The mechanism of why bariatric surgery confers protection from cancer is unknown, but whether this occurs solely from weight loss or other intrinsic changes of the operations remains to be determined ^84^.

## Unanswered questions and future investigation

Bariatric surgery as a treatment for obesity, as well as its benefits on associated chronic medical conditions, continues to gain acceptance and popularity worldwide. Obesity-associated type 2 diabetes as an indication for bariatric surgery is a clear paradigm shift in diabetes management in recent years ^85, 86^. With these clinical changes, a number of important questions continue to arise that are shaping the current and future research landscape. These areas are ripe for investigation and need to be addressed in the coming years for the field of metabolic and bariatric surgery to continue to grow and optimally benefit this increasing patient population.

### Can responders and non-responders be identified preoperatively?

As with many therapies, patient response can vary considerably and this is perhaps most obvious with weight loss following bariatric surgery. From the perspective of weight loss, being able to identify those individuals who will or will not respond is critically important, especially as surgery becomes increasingly used for the treatment of obesity and other diseases. The largest barrier to determining whether we can predict which individuals will respond is the lack of sufficiently large and diverse patient cohorts to be able to construct accurate predictive models. Given the numerous clinical variables of interest (for example, sex, race, baseline body weight, comorbid medical conditions, and operation type), this would require tens of thousands of patients at a minimum. Regardless, smaller retrospective studies have attempted to identify characteristics that suggest success, but these are limited. For example, the higher the baseline BMI, the greater the amount of absolute weight loss in adolescents ^87^ and adults ^88^. Even though this makes practical sense, as individuals with a higher preoperative body weight have much more excess body weight to lose, this finding has not been conclusively demonstrated in adults ^89^.

The largest retrospective analysis (about 27,000 patients), from the Michigan Bariatric Surgery Collaborative ^90^, demonstrated that patients most likely to achieve a BMI of less than 30 kg/m ^2^ were patients who had a preoperative BMI of less than 40 kg/m ^2^. Moreover, these patients had the greatest resolution rates for comorbidities. With respect to diabetes resolution, several studies have similarly suggested that shorter duration of type 2 diabetes and higher preoperative C-peptide concentration are associated with greater diabetes resolution postoperatively ^91^. Along with younger age, which has also been shown to be a positive predictor of better weight loss success ^91, 92^, this suggests that obese diabetic patients benefit from earlier intervention. These findings give credence to the argument that delaying bariatric surgery until individuals reach a BMI of more than 40 kg/m ^2^ may be counterproductive and actually be hurting more people in the end. The effects of withholding effective obesity treatment need to be better examined for the short- and long-term consequences on the patient as well as the health care system ^93^.

The problem with predicting weight loss over time is not straightforward, and being able to predict who will or will not be successful would allow more patient-focused treatment to optimize outcomes for all individuals. However, identifying the patients who will be resistant to surgical weight loss or those individuals who will regain a significant amount of their lost weight over time would be immensely important if those outcomes could be predicted at the initial preoperative consultation. As a corollary, once patients exhibit some degree of weight regain, there is no consensus on how those individuals should be treated ^94^. In most instances, the problem is multifactorial and the solution requires a multidisciplinary approach, although the best strategies for these patients remain unknown. One particularly complicating factor of identifying responders and non-responders is that weight regain may not always be associated with worsening of metabolic endpoints ^95, 96^. Thus, how “failure” of bariatric surgery is defined is critically important to the approach to the patient and overall clinical care.

### Is there a role for metabolic surgery in non-obese patients?

As mentioned, examination of the predictors of who will and will not respond to bariatric surgery has suggested that younger individuals with fewer comorbid medical conditions experience the greatest benefit of bariatric surgery. This raises the question of whether individuals should be receiving bariatric surgery *before* they develop morbid obesity and become generally sicker overall. Bariatric surgery is overall safe and effective at treating diabetes in lower-BMI (<35 kg/m ^2^) individuals ^97^. In fact, clinical data demonstrate significant efficacy of bariatric operations in ethnic groups susceptible to diabetes at lower BMI ranges (<35 kg/m ^2^) ^98– 100^ as well as diabetics without significant obesity ^101– 103^. Clinical and experimental evidence strongly suggests the existence of factors altered by bariatric surgery that drive body weight-independent changes in these patients that are not completely linked to weight loss. The indications for bariatric surgery have changed significantly over the last two decades and paralleled this clinical and experimental evidence. At one time, bariatric surgery was offered only to individuals with a BMI of more than 40 kg/m ^2^. As the benefits of surgical weight loss on obesity-related comorbid diseases became evident, the BMI threshold fell to more than 35 kg/m ^2^ for patients with at least one obesity-related comorbidity. Again, that BMI threshold has fallen, and numerous clinical and professional societies have recently endorsed the consideration of metabolic and bariatric surgery for the treatment of type 2 diabetes in individuals with a BMI in the 30–34.9 kg/m ^2^ range ^85^. Thus, it is reasonable to ask whether or not we should be operating on individuals primarily for intractable diabetes in the absence of obesity.

There is currently insufficient evidence to justify bariatric surgery for non-obese patients, although increasing reports suggest that non-obese diabetics may benefit from bariatric surgery with improved control or resolution of diabetes. Many of these reports are in ethnic groups in which type 2 diabetes develops at a much lower BMI ^104– 106^. We speculate that operating in individuals at lower body weight (25–30 kg/m ^2^) who are at high risk for weight gain and metabolic illness over time may become more commonplace in the coming years. The new clinical guidelines that recommend consideration of “metabolic surgery” in patients with a BMI of less than 35 kg/m ^2^ with intractable diabetes ^85, 86^ are a direct extension of this rationale. Regardless, changes in clinical practice to include individuals with a BMI of less than 30 kg/m ^2^ are not currently supported by any randomized or controlled trials, and any further changes in clinical practice will require further studies.

Aside from the potential health benefits of operating on patients prior to significant weight gain or metabolic illness, bariatric surgery may be associated with decreased health care costs ^107^. However, these cost savings may be easier to realize if individuals undergo bariatric surgery at younger ages when they are less sick and have a better chance of making a full metabolic recovery. Further studies examining these endpoints are needed, but we anticipate that the indications for bariatric and metabolic surgery will continue to broaden with time.

### What is the role of bariatric surgery in adolescent obesity?

The role of bariatric surgery in adolescent obesity is an increasingly debated topic, as the long-term effects of these operations in pediatric patients are largely unknown and understudied. Regardless, pediatric obesity continues to worsen and contributes to the adult obesity epidemic, and it shows no sign of slowing. Even though studies have suggested that treating patients who are younger and not quite as ill from a metabolic standpoint makes sense, the interactions of surgical weight loss with the normal developmental processes in adolescents are unknown. On the contrary, many argue that not offering bariatric surgery is withholding the most effective treatment to a group of adolescents and young adults despite knowing that medical or lifestyle interventions are largely ineffective. Long-term follow-up studies (10–20 years) with close monitoring are necessary in adolescent patients. One such study is under way as part of the Teen-LABS study (Teen-Longitudinal Assessment of Bariatric Surgery), the first observational study of bariatric surgery in adolescents. Teen-LABS enrolled 242 patients undergoing bariatric surgery, which included 161 RYGB and 67 VSG. The 3-year data are promising, showing that weight loss and dyslipidemia markers were improved in all groups ^108^. With the currently unknown long-term effects of bariatric surgery, it is likely that the adolescent population will be targets of increasing obesity treatments in the coming years. Thus, determining the efficacy of these emerging treatments alongside that of bariatric surgery is of utmost importance.

Similar to the adult obesity epidemic, but perhaps more worrisome, is the rise in NAFLD in obese adolescents. NAFLD is expected to only worsen in the coming decade, paralleling the childhood obesity epidemic. Previously, it was thought that pediatric and adolescent patients were protected from developing NAFLD; however, studies have demonstrated that its prevalence is rising. Of the adolescents followed as part of the Teen-LABS study, the largest prospective observational study of adolescent bariatric surgery to date, about 60% had NAFLD at the time of surgery ^109^. Aside from the detrimental medical implications of cirrhosis in an ever-enlarging group of adolescents and young adults ^110, 111^, the economic implications of NAFLD and adolescent obesity are frightening, and these individuals desperately need effective therapies. Longitudinal evaluation of adolescent bariatric surgery is critical in order to identify its efficacy in this population, as surgical management of obesity will likely continue to lag compared with adults.

## Conclusions

Despite the long-held beliefs that obesity is merely a failure of willpower or a character flaw, recent years have proven that body weight regulation, which includes powerful neural controls on appetite and energy expenditure, is much more complex than could ever have been imagined. Although many diseases associated with obesity include those directly related to excess adiposity, including sleep apnea, osteoarthritis, and stress incontinence, other diseases like insulin resistance/diabetes, NAFLD, dyslipidemia, and hypertension appear to be secondary diseases that develop in the chronic inflammatory milieu associated with obesity ^112, 113^.

Metabolic and bariatric surgery for the treatment of obesity and its associated medical conditions is safe overall, and its prevalence undoubtedly will continue to increase in the coming years. With limitations of effective, non-surgical treatment options and continued worsening of the childhood and adult obesity epidemics, it remains to be seen how prevalent obesity surgery may become in younger and less overweight individuals. We speculate that, in the coming decades, the indications for metabolic and bariatric surgery will not only continue to broaden to treat obesity but also preclude its development in high-risk individuals. Further study of the science of bariatric surgery and its profound metabolic effects is critical to increasing the quality of care to this growing patient population as well as combating the economic costs associated with obesity and other metabolic diseases.

## Abbreviations

AGB, adjustable gastric banding; BMI, body mass index; BPD, biliopancreatic diversion; BPD/DS, biliopancreatic diversion with duodenal switch; DSS, Diabetes Surgery Study; LDL, low-density lipoprotein; NAFLD, non-alcoholic fatty liver disease; RYGB, Roux-en-Y gastric bypass; SOS, Swedish Obesity Subjects; STAMPEDE, Surgical Treatment And Medications Potentially Eradicate Diabetes Efficiently; Teen-LABS, Teen-Longitudinal Assessment of Bariatric Surgery; VSG, vertical sleeve gastrectomy
